# Supplementary material for: “You just forget about preeclampsia and move on” –awareness of chronic disease risks and follow-up preferences after preeclampsia in Ireland: a national qualitative study
Source: PLoS One. 2025 Dec 15;20(12):e0337875. doi: 10.1371/journal.pone.0337875 (PMC12704843; doi:10.1371/journal.pone.0337875)
Supplement: S1 File — (DOCX) [file pone.0337875.s001.docx]

**S1. TOPIC GUIDE**

1. **GENERAL DEMOGRAPHIC & OBSTETRIC QUESTIONS**

Thank you for participating in this study. Can I first ask you a few questions about yourself.

1. Your county of residence
2. Year(s) of preeclampsia diagnosis
3. If you had your deliveries in the same county where you live
4. Did you decide to deliver your baby/babies as a public patient or as a private patient?
5. Your age now
6. **PREVIOUS EXPERIENCE OF FOLLOW-UP CARE**

Can you tell me about the follow-up care you received after you were discharged from hospital, if any?

Were you satisfied with your experience of follow-up care?

What went well, or what could have been better?

Do you have any concerns in relation to your follow-up care?

1. **AWARENESS OF LONG-TERM RISKS OF PREECLAMPSIA**

Are you aware of any long-term implications of preeclampsia for your own health?

IF ALREADY AWARE of future health consequences (e.g. raised blood pressure, heart disease, renal disease):

- How did you learn about these health risks?
- Do you think that most women affected by preeclampsia are aware of these health risks?
- What would be your preferred way of learning about any future health consequences?

IF NOT ALREADY AWARE of future health consequences:

- Inform them of the broad associations with raised blood pressure, heart disease, renal disease
- Inform them of opportunities to prevent these chronic diseases through regular monitoring of blood pressure, blood sugar, and kidney function
- Advise them to consider discussing these further with their own GP
- If any concerns are expressed, activate the study Distress Protocol and signpost the participant to the relevant supports available to her.

What would have been your preferred way of learning about any future health consequences?

Do you think that women affected by preeclampsia should be routinely informed of these health risks?

Who do you think should deliver that information to women affected by preeclampsia?

When do you think they should be informed of these health risks?

1. **PREFERENCES REGARDING FOLLOW-UP CARE**

How do you think you, or women like you, should be followed up after preeclampsia?

Would you be interested in enrolling in any structured programme of follow-up care?

IF INTERESTED, what do you think that follow-up care should look like? How/where would you like that follow-up care to be delivered?

IF NOT INTERESTED, would you have other preferences regarding follow-up care after pregnancy?

What do you think might be the benefits of follow-up care for affected women?

Do you think there would be any barriers or disadvantages to receiving follow-up care?

1. **FINAL QUESTIONS**

Do you have any final thoughts on this issue?

Anything you would like to ask about this?

**Thank you very much for your participation in this study.**

**Provide details to get back in touch with Dr. Barrett if desired, or if any further questions.**
