# Supplementary material for: “You just forget about preeclampsia and move on” –awareness of chronic disease risks and follow-up preferences after preeclampsia in Ireland: a national qualitative study
Source: PLoS One. 2025 Dec 15;20(12):e0337875. doi: 10.1371/journal.pone.0337875 (PMC12704843; doi:10.1371/journal.pone.0337875)
Supplement: S2 File — (DOCX) [file pone.0337875.s002.docx]

**S2.** **Selected quotes illustrating responses to questions from topic guide**

| 1. **Previous experience of follow-up care** | | |
| --- | --- | --- |
| ***Can you tell me about the follow-up care you received after you were discharged from hospital, if any?*** | | |
| - | I felt afterwards it was a little bit like, oh, well, you're not pregnant anymore. Good luck! And I kind of got dropped | P5 |
| - | There was no monitoring, there was nothing, it was a bit like, sure you're delivered now, you'll be grand again | P5 |
| - | I never got anything. No info, no other extra care, nothing. | P8 |
| - | there was never any mention of preeclampsia, or the risks, or any follow up, so I never had any follow-up care which for me, looking at my past history now, perhaps, would have been a marker for what I ended up having | P3 |
| - | The shift changed to just my recovery from my Caesarean, and there was very little said about my preeclampsia | P5 |
| - | He told me I had to lose weight just after I had the baby, he said ‘lose a stone’, but you can't do anything other than walking | P8 |
| - | I was advised to, just for my own sake, get a 24 hour blood pressure reading just like, on my own initiative | P9 |
| - | I couldn't complain about anything. They were all so good the whole journey through | P12 |
| ***Do you have any concerns in relation to your follow-up care?*** | | |
| - | There was no communication between the hospital and the GP which made it quite challenging | P9 |
| - | The connection between the obstetrician and the GP needs to be better.  Or maybe the obstetrician even needs to be educated about the fact that there is this follow up programme in place | P9 |
| - | I don't know how much your maternity hospital links in really with your GP. But if there's some way of advising them that you've had preeclampsia, or something like that that they noticed as well. Because, you know, you're the one imparting all those details to your GP afterwards. So if there was some way that that was linked up, that would be great. | P6 |
| 1. **Awareness of long-term risks after preeclampsia** | | |
| ***Are you aware of any long-term implications of preeclampsia for your own health?*** | | |
| - | No. I was never, I was never really aware that there was any long term effects. I thought maybe it was just something that was associated with your pregnancy, and went away thereafter. But I I don't really know anything else about it | P6 |
| - | The only thing I was told was, the minute that baby comes out, you're fine. That was it, and I was like, oh, it's that easy, is it? | P4 |
| - | Never gave it a second thought. To be honest | P10 |
| - | The only info I ever got was one midwife, after my son was born, who said just to let you know that, like, once you've had preeclampsia once, you might be predisposed to high blood pressure when you're after menopause | P8 |
| - | I suppose more the very long term is something I would have found out myself. Cardiovascular and stroke kind of thing. So nobody really specifically sat down and said you are a little bit more at risk of this or this. | P12 |
| ***How did you learn about these health risks? (If already aware)*** | | |
| - | It's nothing that I've been told by a doctor or anything. It's more from what I've read online and on my own research. | P1 |
| - | It is a bit annoying that I have to go and look and find out myself, that there isn't any sort of clinic or specialist or someone that you can go and sit down with and talk out your concerns or be sat down and told. | P1 |
| - | The only reason I know that is because I've Googled stuff, you know. No doctor has ever mentioned it to me, but I mean, like I know it from what I've seen online . | P11 |
| - | Google was my friend. And you know sometimes Google is really not the friend you want, because it sends you down the worst case scenario | P4 |
| ***What would be your preferred way of learning about any future health consequences?*** | | |
| - | It would be lovely if it was kind of a verified information supported by the people who had cared for you | P2 |
| - | I think there's so much information on the HSE website I I found it really beneficial in the initial few weeks after having our daughter that if there was maybe a platform or a place to go that would lead you to where you could ask the questions | P2 |
| - | If I have something concrete in my hand, you know you could stick it up on the fridge , or something like that, just as ever, you know, as a reminder. | P11 |
| - | It could easily be put into a pack somewhere of all the stuff they send you off with. You know it's not hard to add it in, just to flag it as an issue that you can keep up with your GP | P6 |
| - | I want total transparency. Give me the good and the bad and the ugly | P4 |
| - | Having the information about the risks, but the modifiable nature of them, would be the important thing | P7 |
| - | If you're made aware of it, that it can affect you going forward, I think that would plant the seed in your brain that you want to maybe get it checked out further down the line. | P6 |
| - | I suppose, in one respect, being ignorant of the fact, probably helped me | P3 |
| ***Who do you think should deliver that information to women affected by preeclampsia?*** | | |
| - | Your GP, just to give you the information on it. I mean, most people have a good relationship with their GPs anyway. | P10 |
| - | I mean if it was the GP I would have been fine with that. Again, the public health nurse, because you're with them with your baby. | P2 |
| - | I would say probably best is the GP practice or… you know obviously they're doing all the chronic disease management and everything | P12 |
| - | Public health nurse could be a good one ’cause you do have a review with them like nine or ten months or something. | P8 |
| ***When do you think they should be informed of these health risks?*** | | |
| - | You're not in the headspace for it, I think for probably 6 to 12 weeks. | P8 |
| - | Obviously in my situation at that time when I did see the doctors again (shortly after birth), no, that definitely wouldn't have been the right time | P11 |
| - | I remember being bombarded with so much stuff in the hospital and the neonatal unit and everything . I don't think it would have been top of my priority | P10 |
| - | I would love to think that, maybe even a few months down the line when you're over the chaos of a new baby, that there is an opportunity to have a discussion and just to learn a little bit more about it | P2 |
| 1. **Preferences regarding follow-up care** | | |
| ***What do you think that follow-up care should look like? How/where would you like that follow-up care to be delivered?*** | | |
| - | Definitely one-to-one person human contact , you know, someone that could even just empathise | P4 |
| - | If there was something that would, you know… there was a default, saying, here's your appointment to come in and see us, that would be fantastic | P6 |
| - | If an effort has been made from someone else's part to make sure that my health is OK. Yeah, it kind of helps you to almost prioritise it then | P1 |
| - | In GP care, but trying to get an appointment… even a GP now is unbelievable. | P1 |
| - | Whereas city center hospitals, traffic, parking, everything. No, I I'd be too stressed at the thought of having to get in and find parking in town, so I'd be just like, I'm not going to it, thanks | P7 |
| - | If it was flagged on women's file even in GP, that they had preeclampsia, you know, so we'll say you are 15 years down the line, and you're at the GP for something, it should still say preeclampsia on the file, so that the GP will be thinking … we’ll just check your blood pressure | P8 |
| - | You don't have to go into a hospital, because, like the, you know, an awful lot of women who've had preeclampsia like they'll be traumatised. | P11 |
| ***Do you think there would be any barriers or disadvantages to receiving follow-up care?*** | | |
| - | I guess people, maybe, who might have language barriers and that kind of thing would perhaps be difficult for them | P1 |
| - | As soon as I see the blood pressure cuff, my heart starts racing, and this is 15 years later, and it still happens, so there could be women who would actually just not want to do that, they would want to avoid it for that reason | P10 |
| - | She was like, well, you need to get a monitor, and I was like, I don't have a hundred quid spare right now, because I didn't. We didn't have two pennies to rub together | P5 |
| - | I do definitely think it should be public funded because obviously like financial strains and everyone | P1 |
| - | Probably cost would be something that would come into things as well, but I suppose if it's going to be a public thing… it's going to be taken up a lot better. | P12 |
| - | There's so much more involved than just simply getting into your car and going to the appointment . There might be lots of other factors for them to consider. Yeah, childcare and logistics and everything else. | P4 |
| - | Just because it's, you know, it's logistics behind it, going into Dublin, whether you have a small baby that you have to carry with you like. I brought him all the time with me to the appointments, or I had to get my husband to mind the kids and if you're working like, you know, you have to take time off. | P9 |
